# Causal inference of asynchronous audiovisual speech

**DOI:** 10.3389/fpsyg.2013.00798

**Published:** 2013-11-13

**Authors:** John F. Magnotti, Wei Ji Ma, Michael S. Beauchamp

**Affiliations:** ^1^Department of Neurobiology and Anatomy, University of Texas Medical School at HoustonTX, USA; ^2^Department of Neuroscience, Baylor College of MedicineHouston TX, USA

**Keywords:** causal inference, synchrony judgments, speech perception, multisensory integration, Bayesian observer

## Abstract

During speech perception, humans integrate auditory information from the voice with visual information from the face. This multisensory integration increases perceptual precision, but only if the two cues come from the same talker; this requirement has been largely ignored by current models of speech perception. We describe a generative model of multisensory speech perception that includes this critical step of determining the likelihood that the voice and face information have a common cause. A key feature of the model is that it is based on a principled analysis of how an observer should solve this causal inference problem using the asynchrony between two cues and the reliability of the cues. This allows the model to make predictions about the behavior of subjects performing a synchrony judgment task, predictive power that does not exist in other approaches, such as *post-hoc* fitting of Gaussian curves to behavioral data. We tested the model predictions against the performance of 37 subjects performing a synchrony judgment task viewing audiovisual speech under a variety of manipulations, including varying asynchronies, intelligibility, and visual cue reliability. The causal inference model outperformed the Gaussian model across two experiments, providing a better fit to the behavioral data with fewer parameters. Because the causal inference model is derived from a principled understanding of the task, model parameters are directly interpretable in terms of stimulus and subject properties.

## Introduction

**Figure 1:**
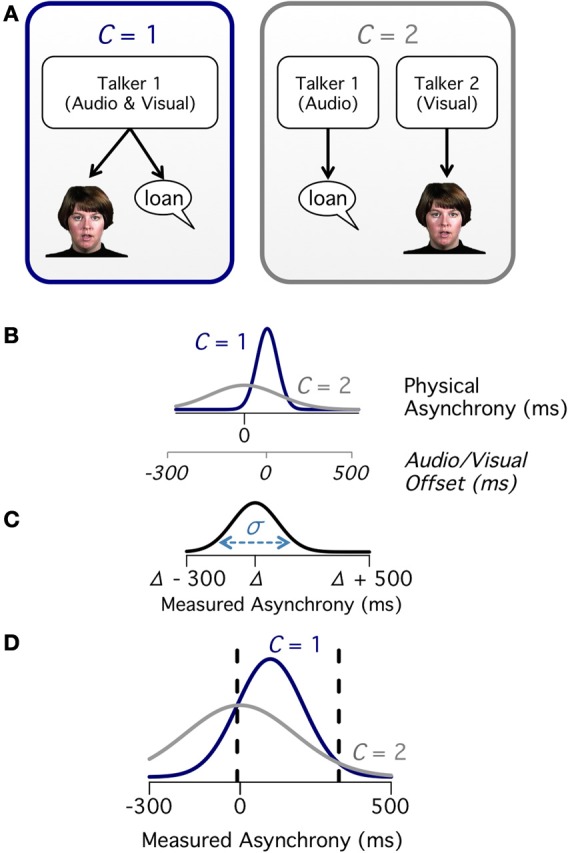
**Causal structure of audiovisual speech. (A)** Causal diagram for audiovisual speech emanating from a single talker (*C* = 1) or two talkers (*C* = 2). **(B)** Difference between auditory and visual speech onsets showing a narrow distribution for *C* = 1 (navy) and a broad distribution for *C* = 2 (gray). The first x-axis shows the onset difference in the reference frame of physical asynchrony. The second x-axis shows the onset difference in reference frame of the stimulus (audio/visual offset created by shifting the auditory speech relative to the visual speech). A recording of natural speech without any manipulation corresponds to zero offset in the stimulus reference frame and a positive offset in the physical asynchrony reference frame because visual mouth opening precedes auditory voice onset. **(C)** For any given physical asynchrony (Δ) there is a distribution of measured asynchronies (with standard deviation σ) because of sensory noise. **(D)** Combining the likelihood of each physical asynchrony **(B)** with sensory noise **(C)** allows calculation of the measured asynchrony distributions across all physical asynchronies. Between the dashed lines, the likelihood of *C* = 1 is greater than the likelihood of *C* = 2.

When an observer hears a voice and sees mouth movements, there are two potential causal structures (Figure 1A). In the first causal structure, the events have a common cause (*C* = 1): a single talker produces the voice heard and the mouth movements seen. In the second causal structure, the events have two different causes (*C* = 2): one talker produces the auditory voice and a different talker produces the seen mouth movements. When there is a single talker, integrating the auditory and visual speech information increases perceptual accuracy (Sumby and Pollack, 1954; Rosenblum et al., 1996; Schwartz et al., 2004; Ma et al., 2009); most computational work on audiovisual integration of speech has focused on this condition (Massaro, 1989; Massaro et al., 2001; Bejjanki et al., 2011). However, if there are two talkers, integrating the auditory and visual information actually *decreases* perceptual accuracy (Kording et al., 2007; Shams and Beierholm, 2010). Therefore, a critical step in audiovisual integration during speech perception is estimating the likelihood that the speech arises from a single talker. This process, known as causal inference (Kording et al., 2007; Schutz and Kubovy, 2009; Shams and Beierholm, 2010; Buehner, 2012), has provided an excellent tool for understanding the behavioral properties of tasks requiring spatial localization of simple auditory beeps and visual flashes (Kording et al., 2007; Sato et al., 2007). However, multisensory speech perception is a complex and highly-specialized computation that takes place in brain areas distinct from those that perform audiovisual localization (Beauchamp et al., 2004). Unlike spatial localization, in which subjects estimate the continuous variable of location, speech perception is inherently multidimensional (Ma et al., 2009) and requires categorical decision making (Bejjanki et al., 2011). Therefore, we set out to determine whether the causal inference model could explain the behavior of humans perceiving multisensory speech.

Manipulating the asynchrony between the auditory and visual components of speech dramatically affects audiovisual integration. Therefore, synchrony judgment tasks are widely used in the audiovisual speech literature and have been used to characterize both individual differences in speech perception in healthy subjects and group differences between healthy and clinical populations (Lachs and Hernandez, 1998; Conrey and Pisoni, 2006; Smith and Bennetto, 2007; Rouger et al., 2008; Foss-Feig et al., 2010; Navarra et al., 2010; Stevenson et al., 2010; Vroomen and Keetels, 2010). While these behavioral studies provide valuable descriptions of behavior, the lack of a principled, quantitative foundation is a fundamental limitation. In these studies, synchrony judgment data are fit with a series of Gaussian curves without a principled justification for why synchrony data should be Gaussian in shape. A key advantage of the causal inference model is that the model parameters, such as the sensory noise in each perceiver, can be directly related to the neural mechanisms underlying speech perception. This stands in sharp contrast to the Gaussian model's explanations based solely on descriptive measures (most often the standard deviation of the fitted curve). The causal inference model generates behavioral predictions based on an analysis of how the brain might best perform the task, rather than seeking a best-fitting function for the behavioral data.

## Causal inference of asynchronous audiovisual speech

The core of the causal inference model is a first-principles analysis of how the relationship between cues can be used to determine the likelihood of a single talker or multiple talkers. Natural auditory and visual speech emanating from the same talker (*C* = 1) contains a small delay between the visual onset and the auditory onset caused by the talker preparing the facial musculature for the upcoming vocalization before engaging the vocal cords. This delay results in the distribution of asynchronies having a positive mean when measured by the physical difference between the auditory and visual stimulus onsets (Figure 1B). When there are two talkers (*C* = 2), there is no relationship between the visual and auditory onsets, resulting in a broad distribution of physical asynchronies. Observers do not have perfect knowledge of the physical asynchrony, but instead their measurements are subject to sensory noise (Figure 1C). An observer's measured asynchrony therefore follows a distribution that is broader than the physical asynchrony distribution. Overlaying these distributions shows that there is a window of measured asynchronies for which *C* = 1 is more likely than *C* = 2 (Figure 1D). This region is the Bayes-optimal synchrony window and is used by the observer to make the synchronous/asynchronous decision; this window does not change based on the physical asynchrony, which is unknown to the observer.

**Figure 2:**
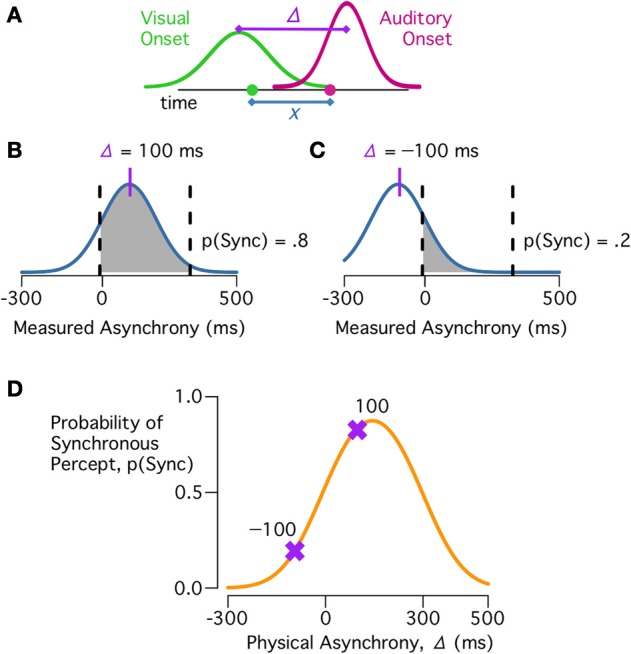
**Synchrony judgments under the causal inference model. (A)** On each trial, observers obtain a measurement of the audiovisual asynchrony by differencing measurements of the auditory (magenta) and visual (green) onsets. Because of sensory noise, the measured asynchrony (*x*, blue) is different than the physical asynchrony (Δ, purple). **(B)** For a given physical asynchrony, Δ = 100 ms, there is a range of possible measured asynchronies (*x*, blue). The shaded region indicates values of *x* for which *C* = 1 is more probable than *C* = 2 (Figure 1D). The area of the shaded region is the probability of a synchronous percept, p(Sync). **(C)** For a different physical asynchrony, Δ = −100 ms, there is a different distribution of measured asynchronies, with a lower probability of a synchronous percept. **(D)** The probability of a synchronous percept for different physical asynchronies. Purple markers show the predictions for Δ = 100 ms and Δ = −100 ms.

During perception of a multisensory speech event, the measured onsets for the auditory and visual cues are corrupted by sensory noise (Ma, 2012); these measurements are subtracted to produce the measured asynchrony (*x*; Figure 2A). Critically, observers use this measured asynchrony, rather than the physical asynchrony, to infer the causal structure of the speech event. Because the sensory noise has zero mean, the physical asynchrony determines the mean of the measured asynchrony distribution. Thus, synchrony perception depends both on the physical asynchrony and the observer's sensory noise. For example, if the visual cue leads the auditory cue by 100 ms (physical asynchrony = 100 ms, the approximate delay for cues from the same talker), then the measured asynchrony is likely to fall within the Bayes-optimal synchrony window, and the observer is likely to respond synchronous (Figure 2B). By contrast, if the visual cue trails the auditory cue by 100 ms (physical asynchrony = −100 ms) then the measured asynchrony is unlikely to fall within the synchrony window and the observer is unlikely to report a common cause (Figure 2C). Calculating the likelihood that a measured asynchrony falls within the synchrony window for each physical asynchrony at a given level of sensory noise produces the predicted behavioral curve (Figure 2D). Because the sensory noise is modeled as changing randomly from trial-to-trial, the model is probabilistic, calculating the probability of how an observer will respond, rather than deterministic, with a fixed response for any physical asynchrony (Ma, 2012).

## Materials and methods

### Behavioral testing procedure

Human subjects approval and subject consent were obtained for all experiments. Participants (*n* = 39) were undergraduates at Rice University who received course credit. All participants reported normal or corrected-to-normal vision and hearing.

Stimuli were presented on a 15″ Macbook Pro Laptop (2008 model) using Matlab 2010a with the Psychophysics Toolbox extensions (Brainard, 1997; Pelli, 1997) running at 1440 × 900 (width × height) resolution. Viewing distance was ~40 cm. A lamp behind the participants provided low ambient lighting. Sounds were presented using KOSS UR40 headphones. The volume was set at a comfortable level for each individual participant.

Trials began with the presentation of a white fixation cross in a central position on the screen for 1.2 s, followed by presentation of the audiovisual recording of a single word (~2 s), and then the reappearance of the fixation cross until the behavioral response was recorded. Participants were instructed to press the “m” key if the audio and visual speech were perceived as synchronous, and the “n” key if perceived as asynchronous.

### Stimuli

The stimuli consisted of audiovisual recordings of spoken words from previous studies of audiovisual speech synchrony judgments (Lachs and Hernandez, 1998; Conrey and Pisoni, 2004) obtained by requesting them from the authors. The stimuli were all 640 × 480 pixels in size. The first stimulus set consisted of recordings of four words (“doubt,” “knot,” “loan,” “reed”) selected to have high visual intelligibility, as determined by assessing visual-only identification performance (Conrey and Pisoni, 2004). The temporal asynchrony of the auditory and visual components of the recordings was manipulated, ranging from −300 ms (audio ahead) to +500 ms (video ahead), with 15 total asynchronies (−300, −267, −200, −133, −100, −67, 0, 67, 100, 133, 200, 267, 300, 400, and 500 ms). The visual-leading half of the curve was over-sampled because synchrony judgment curves have a peak shifted to the right of 0 (Vroomen and Keetels, 2010). The second stimulus set contained blurry versions of these words (at the same 15 asynchronies), created by blurring the movies with a 100-pixel Gaussian filter using FinalCut Pro. For the third stimulus set, four words with low visual intelligibility were selected (“give,” “pail,” “theme,” “voice”) at the same 15 asynchronies. The fourth stimulus set contained visual-blurred versions of the low visual intelligibility words.

### Experimental design

For the first experiment, stimuli from all four stimulus sets were presented, randomly interleaved, to a group of 16 subjects in one testing session for each subject. The testing session was divided into three blocks, with self-paced breaks between each run. Each run contained one presentation of each stimulus (8 words × 15 asynchronies × 2 reliabilities = 240 total stimuli per run). Although the stimulus sets were presented intermixed, and a single model was fit to all stimuli together, we discuss the model predictions separately for each of the four stimulus sets.

In the second, replication experiment, the same task and stimuli were presented to a group of 23 subjects (no overlap with subjects from Experiment 1). These subjects completed one run, resulting in one presentation of each stimulus to each subject. Two subjects responded “synchronous” to nearly all stimuli (perhaps to complete the task as quickly as possible). These subjects were discarded, leaving 21 subjects.

### Model parameters

The causal inference of multisensory speech (CIMS) model has two types of parameters: two subject parameters and four stimulus parameters (one of which is set to zero, resulting in three fitted parameters). The first subject parameter, σ, is the noise in the measurement of the physical asynchrony (while we assume that the measurement noise is Gaussian, other distributions could be used easily), and varies across stimulus conditions. Because the noisy visual and noisy auditory onset estimates are differenced, only a single parameter is needed to estimate the noise in the measured asynchrony. As σ increases, the precision of the observer's measurement of the physical asynchrony decreases.

The second subject parameter is *p*_*C* = 1_, the prior probability of a common cause. This parameter is intended to reflect the observer's expectation of how often a “common cause” occurs in the experiment, and remains fixed across stimulus conditions. Holding other parameters constant, a higher *p*_*C* = 1_ means that the observer will more often report synchrony. If observers have no systematic bias toward *C* = 1 or *C* = 2, we have *p*_*C* = 1_ = 0.5, and the model has only one subject-level parameter.

The four stimulus parameters reflect the statistics of natural speech and are the mean and standard deviation of the *C* = 1 (μ_*C* = 1_, σ_*C* = 1_) and *C* = 2 distributions (μ_*C* = 2_, σ_*C* = 2_).

### Physical asynchrony vs. auditory/visual offset

In the literature, a common reference frame is to consider the manipulations made to speech recordings when generating experimental stimuli (Vroomen and Keetels, 2010). In this convention, natural speech is assigned an audio/visual offset of zero. These two reference frames (physical vs. stimulus manipulation) differ by a small positive shift that varies slightly across different words and talkers. This variability in the *C* = 1 distribution is accounted for in the model with a narrow distribution of physical asynchronies. Talkers consistently begin their (visually-observed) mouth movements slightly before beginning the auditory part of the vocalization. This results in a narrow asynchrony distribution for *C* = 1 (small σ_*C* = 1_). In contrast, if the visually-observed mouth movements arise from a different talker than the auditory vocalization, we expect no relationship between them and a broad asynchrony distribution for *C* = 2 (large σ_*C* = 2_). We adopt the widely-used stimulus manipulation reference frame and define μ_*C* = 1_ as zero, which leads to a negative value for μ_*C* = 2_ (adopting the other reference frame does not change the results).

### Model fitting and comparison

All model fitting was done in R (R Core Team, 2012). The source code for all models is freely available on the authors' web site (http://openwetware.org/wiki/Beauchamp:CIMS). Only a single model was fit for each subject across all stimulus conditions. The input to the model fitting procedures was the number of times each physical asynchrony was classified as synchronous across all runs. All model parameters were obtained via maximization of the binomial log-likelihood function on the observed data.

For the CIMS model, we used a multi-step optimization approach. In the first step, we found the best-fitting subject parameters (*p*_*C* = 1_ and σ) for each subject and stimulus set based on random initial values for the stimulus parameters (σ_*C* = 1_, σ_*C* = 2_, μ_*C* = 2_). In the second step, we found the best-fitting stimulus parameters based on the fitted *p*_*C* = 1_ and σ values. These steps were repeated until the best-fitting model was obtained. Because the experimental manipulations were designed to affect sensory reliability, we fit a separate σ in each condition, resulting in a total of 8 free parameters for the CIMS model. This hierarchical fitting procedure was used because some parameters were consistent across conditions, allowing the fitting procedure to converge on the best fitting model more quickly. We refit the model using 256 initial positions for the stimulus parameters to guard against fitting to local optima. Visual inspection of individual fits confirmed the model was not fitting obviously sub-optimal subject parameters. Finally, we confirmed the ability of the fitting procedure to recover the maximum likelihood estimates for each parameter using simulated data.

We compared the CIMS model with a curve-fitting approach taken in previous studies of audiovisual synchrony judgments, which we term the Gaussian model. In the Gaussian model, a scaled Gaussian probability density curve is fit to each subject's synchrony judgment curve. Each subject's synchrony judgment curve is therefore characterized by the three parameters: the mean value, the standard deviation, and a scale parameter that reflects the maximum rate of synchrony perception. Because the Gaussian model does not make any predictions about the relationship between conditions, we follow previous studies and fit independent sets of parameters between stimulus sets. The Gaussian model contains a total of 12 free parameters across all conditions in this experiment.

After fitting the models to the behavioral data, we compared them based on how well their predictions matched the observed data. We show the mean model predictions with the mean behavioral data to assess qualitative fit. Because these predicted data are averages, however, they are not a reasonable indicator of a model's fit to any individual subject. A model that overpredicts synchrony for some subjects and underpredicts for others may have a better mean curve than a model that slightly overpredicts more often than underpredicts. To ensure the models accurately reproduce individual-level phenomena, we assess model fit by aggregating error across individual-level model fits. In experiment 1 we provide a detailed model comparison for each stimulus set using the Bayesian Information Criterion (BIC). BIC is a linear transform of the negative log-likelihood that penalizes models based on the number of free parameters and trials, with lower BIC values corresponding to better model fits. For each model we divide the penalty term evenly across the conditions, so that the CIMS model is penalized for 2 parameters per condition and the Gaussian model is penalized for 3 parameters per condition. To compare model fits across all stimulus conditions we considered both individual BIC across all conditions and the group mean BIC, calculated by summing the BIC for each condition across subjects, then taking the mean across subjects. In Experiment 2, we compare group mean BIC across all conditions. Conventional significance tests on the BIC differences between models were also performed. In the model comparison figures, error is the within-subject standard error, calculated as in Loftus and Masson (1994).

### Model derivation

In the generative model, there are two possible states of the world: *C* = 1 (single talker) and *C* = 2 (two talkers). The prior probability of *C* = 1 is *p*_*C* = 1_. Asynchrony, denoted Δ, has a different distribution under each state. Both are assumed to be Gaussian, such that *p*(Δ|*C* = 1) = *N* (Δ; 0, σ^2^_1_) and *p*(Δ|*C* = 2) = *N* (Δ; μ_2_, σ^2^_2_). The observer's noisy measurement of Δ, denoted *x*, is also Gaussian, *p*(*x*|Δ) = *N*(*x*;Δ, σ^2^) where the variance σ^2^ is the combined variance from the auditory and visual cues. This specifies the statistical structure of the task. In the inference model, the observer infers *C* from *x*. This is most easily expressed as a log posterior ratio, $d=\log\frac{p(C=1|x)}{p(C=2|x)}=\log\frac{p_{C=1}}{1-p_{C=1}}+\log\frac{N\left(x;0,\sigma^{2}+\sigma_{1}^{2}\right)}{N\left(x;\mu ,\sigma^{2}+\sigma_{2}^{2}\right)}$.

The optimal decision rule is *d* > 0. If we assume that σ_1_ < σ_2_, then the optimal decision rule becomes $\left|x+\mu_{2}\frac{\sigma^{2}+\sigma_{2}^{2}}{\sigma_{2}^{2}-\sigma_{1}^{2}}\right|<\sqrt{\frac{2\log\frac{p_{C=1}}{1-p_{C=1}}+\log\frac{\sigma^{2}+\sigma_{2}^{2}}{\sigma^{2}+\sigma_{1}^{2}}+\frac{\mu_{2}^{2}}{\sigma_{2}^{2}-\sigma_{1}^{2}}}{\left(\frac{1}{\sigma^{2}+\sigma_{1}^{2}}-\frac{1}{\sigma^{2}+\sigma_{2}^{2}}\right)}}$ and the probability of reporting a common cause for a given asynchrony is $p(\hat{C}=1|\Delta )=\text{Normcdf}\left(x;-\mu_{2}\frac{\sigma^{2}+\sigma_{2}^{2}}{\sigma_{2}^{2}-\sigma_{1}^{2}}+\sqrt{\ldots ,}\sigma \right)-\text{Normcdf}\left(x;-\mu_{2}\frac{\sigma^{2}+\sigma_{2}^{2}}{\sigma_{2}^{2}-\sigma_{1}^{2}}-\sqrt{\ldots ,}\sigma \right)$.

## Results

### Behavioral results from experiment 1

The CIMS model makes trial-to-trial behavioral predictions about synchrony perception using a limited number of parameters that capture physical properties of speech, the sensory noise of the subject, and the subject's prior assumptions about the causal structure of the stimuli in the experiment. The Gaussian model simply fits a Gaussian curve to the behavioral data. We tested the CIMS model and Gaussian model against behavioral data from subjects viewing movie clips of audiovisual speech with varying asynchrony, visual intelligibility (high/low) and visual reliability (reliable/blurred).

#### Visual reliable, high visual intelligibility words

**Figure 3:**
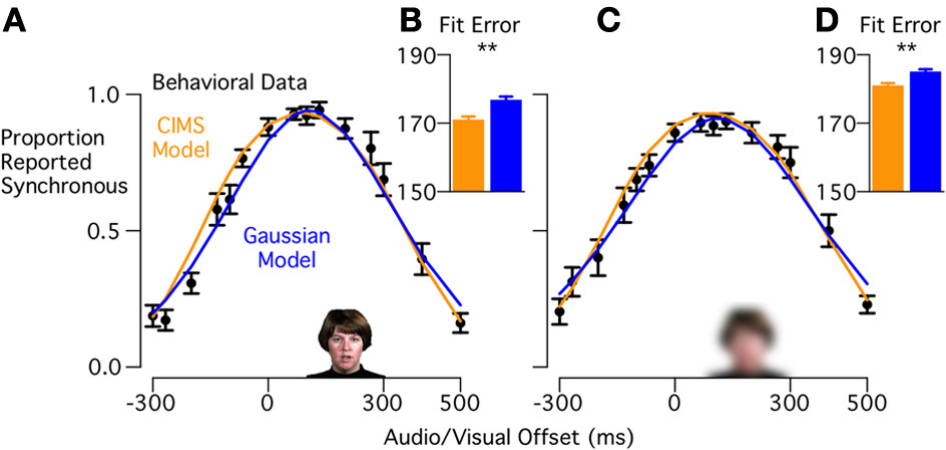
**Model fits to behavioral data for experiment 1 (high visual intelligibility words). (A)** Black circles show the behavioral data from 16 subjects performing a synchrony judgment task (mean ± standard error) for each stimulus asynchrony with visual reliable, high visual intelligibility stimuli. Curves show the model predictions for the CIMS model (orange) and Gaussian model (blue). **(B)** Fit error measured with Bayesian Information Criterion (BIC) for the CIMS and Gaussian models; lower values indicate a better fit for the CIMS model (^**^p = 0.006). Error bars show within-subject standard error (Loftus and Masson, 1994). **(C)** Mean proportion of synchrony responses and model predictions for visual blurred, high visual intelligibility stimuli. **(D)** Fit error for the CIMS and Gaussian models, showing better fit for the CIMS model (^**^p = 0.007).

We first compared synchrony judgments for 16 subjects with visual reliable, high visual intelligibility words. Synchronous responses were 0.90 or higher for visual-leading asynchronies from +67 to +133 ms, but dropped off for higher or lower asynchronies (Figure 3A). The general shape of the curve is consistent with previous reports of simultaneity judgments with these stimuli (Conrey and Pisoni, 2006).

A single CIMS model and Gaussian model were fit to each subject across all stimulus conditions and the predicted synchrony reports were averaged to produce mean predictions (Figure 3A). To provide a quantitative comparison of the model fits, we compared the BIC of both models for each subject (Figure 3B). The BIC measure was in favor of the CIMS model, with a mean difference of 5.8 ± 1.8 (SEM). A paired *t*-test showed that the difference was reliable [*t*_(15)_ = 3.16, *p* = 0.006].

The better fit of the CIMS models is caused by its ability to predict a range of asynchronies that are perceived as nearly synchronous (rather than one peak) and an asymmetric synchrony judgment curve. These features are consequences of the model structure, not explicit parameters of the model. The presence of noise in the sensory system means that even when the physical asynchrony is identical to the mean of the common cause distribution there is still a chance the measured asynchrony will be outside the synchrony window. Having an asymmetric, broad range of synchronies reported as nearly synchronous is predicted by the interaction of the observer's prior belief about the prevalence of a common cause in this experiment and sensory noise.

#### Visual blurred, high visual intelligibility words

Because blurring decreases the reliability of the visual speech, the CIMS model predicts that the sensory noise level should increase, resulting in changes in synchrony perception primarily at larger asynchronies. Despite the blurring, the peak of the synchrony judgment curve remained high (around 0.9 reported synchrony for +67 to +133 ms) showing that participants were still able to perform the task. However, the distribution had a flatter top, with participants reporting high synchrony values for a broad range of physical asynchronies that extended from 0 ms (no audio/visual offset) to +267 ms (Figure 3C). The drop-off in reported synchrony was more asymmetric than for unblurred stimuli, dropping more slowly for the visual-leading side of the curve. Comparing the model fits to the behavioral data, the CIMS model was supported (Figure 3D) over the Gaussian model [BIC difference: 4.1 ± 1.3, *t*_(15)_ = 3.1, *p* = 0.007]. Blurring the visual speech makes estimation of the visual onset harder by adding uncertainty to the observer's estimate of the visual onset. For the CIMS model, this has the effect of increasing variability, leading to a widening of the predicted behavioral curves and an exaggeration of their asymmetry. In contrast, for the Gaussian model, increasing the standard deviation can only symmetrically widen the fitted curves.

#### Visual reliable, low visual intelligibility words

**Figure 4:**
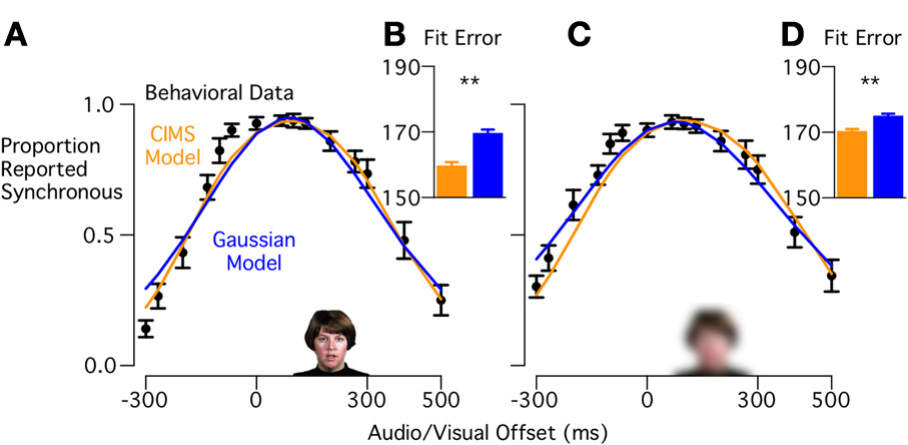
**Model fits to behavioral data for Experiment 1 (low visual intelligibility words). (A)** Black circles show the behavioral data with visual reliable, low visual intelligibility stimuli, curves show model predictions for CIMS (orange) and Gaussian (blue) models. **(B)** Fit error showing significantly better fit for the CIMS model (^**^p < 0.001). **(C)** Mean proportion of synchrony responses and model predictions for visual blurred, low visual intelligibility stimuli. **(D)** Fit error showing significantly better fit for the CIMS model (^**^p = 0.001).

In the CIMS model, words with low visual intelligibility should decrease the certainty of the visual speech onset, corresponding to an increase in sensory noise. Decreasing visual intelligibility both widened and flattened the peak of the synchrony judgment curve (Figure 4A), resulting in a broad plateau from −67 ms to +133 ms. When the CIMS and Gaussian model fits were compared (Figure 4B), the CIMS model provided a better fit to the behavioral data [BIC difference: 9.9 ± 2.0, *t*_(15)_ = 4.9, *p* < 0.001]. The CIMS model accurately predicts the plateau observed in the behavioral data, in which a range of small asynchronies are reported as synchronous with high probability. The Gaussian model attempts to fit this plateau through an increased standard deviation, but this resulted in over-estimating synchrony reports at greater asynchronies.

#### Visual blurred, low visual intelligibility words

The CIMS model predicts that visual blurring should be captured solely by a change in sensory noise. Blurring led to an increase in synchrony reports primarily for the larger asynchronies (Figure 4C). The height and location of the plateau of the curve was similar to the unblurred versions of these words (between 0.89 and 0.93 synchrony reports from −67ms to +133 ms). Overall, blurring the low visual intelligibility words had generally the same effect as blurring the high visual intelligibility words: widening the synchrony judgment curve, but not changing the height or position of the curve's plateau. The CIMS model fit the behavioral data better (Figure 4D) than the Gaussian model [BIC difference: 4.7 ± 1.2, *t*_(15)_ = 3.9, *p* = 0.001]. The better fit resulted from the CIMS model's ability to predict an asymmetric effect of blurring and continued prediction of a wide range of asynchronies reported as synchronous with high probability.

#### Overall model testing

**Figure 5:**
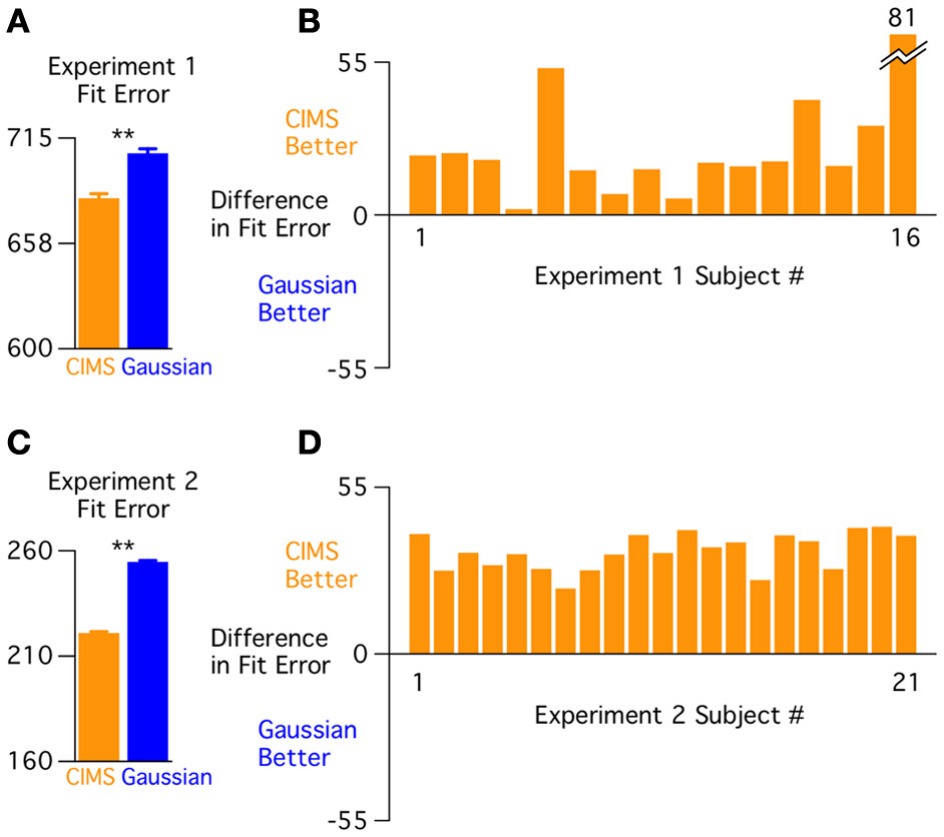
**Model comparison across experiments. (A)** Total fit error (Bayesian Information Criterion; BIC) across conditions averaged over the 16 subjects in experiment 1 showing better fit for the CIMS model (^**^p < 0.001). Error bars are within-subject standard error (Loftus and Masson, 1994). **(B)** Difference in fit error (BIC) for each individual subject (across all conditions). **(C)** Total fit error across conditions averaged over the 21 subjects in experiment 2 showing better fit for the CIMS model (^**^p < 10^−15^). **(D)** Difference in fit error for each individual subject (across all conditions).

Next, we compared the models across all stimulus sets together. For the CIMS model, the parameters σ_*C* = 1_, σ_*C* = 2_, μ_*C* = 2_, *p*_*C* = 1_ for each subject are fit across all conditions, placing constraints on how much synchrony perception can vary across conditions (the Gaussian model has no such constraints because a separate scaled Gaussian is fit for each condition). We compared the models across all conditions using the average of the total BIC (summed across stimulus sets) across subjects (Figure 5A). Despite the additional constraints of the CIMS model, the overall model test supported it over the Gaussian model [BIC difference: 24.5 ± 4.9, *t*_(15)_ = 4.99, *p* < 0.001]. The direction of this difference was replicated across all 16 subjects (Figure 5B), although the magnitude showed a large range (range of BIC differences: 2–81).

It is tempting to compare the fit error across different stimulus conditions within each model to note, for instance, that the fit error for visual blurring is greater than without it; or that the models fit better with low visual intelligibility words. However, these comparisons must be made with caution because the stimulus level parameters are fit across all conditions simultaneously and are thus highly dependent. For instance, removing the visual intelligibility manipulation would change the parameter estimates and resulting fit error for the visual blurring condition. The only conclusion that can be safely drawn is that the CIMS model provides a better fit than the Gaussian model for all tested stimulus manipulations.

#### Interpreting parameters from the CIMS model

**Figure 6:**
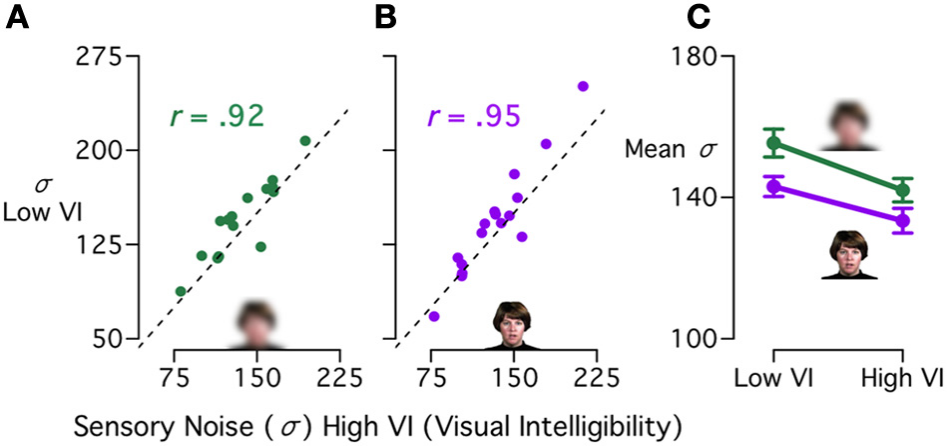
**Model estimates of sensory noise across stimuli in experiment 1. (A)** Correlation between CIMS model sensory noise (σ) estimates for visual blurred words with high visual intelligibility (high VI) and low VI. Each symbol represents one subject, the dashed line indicates equal sensory noise between the two conditions. There was a strong positive correlation (*r* = 0.92, *p* < 10^−6^). **(B)** Correlation between sensory noise estimates for visual reliable words with high and low VI (*r* = 0.95, *p* < 10^−7^). **(C)** Mean sensory noise across subjects [± within-subject standard error of the mean (Loftus and Masson, 1994)] for visual blurred words (green line) and visual reliable words (purple line) with low VI (left) or high VI (right).

A key property of the CIMS model is the complete specification of the synchrony judgment task structure, so that model parameters may have a meaningful link to the cognitive and neural processes that instantiate them. First, we examined how σ, the sensory noise parameter, changed across stimulus conditions and subjects (Figure 6).

The fitted value of σ is a measure of the sensory noise level in each individual and condition, and captures individual differences in the task. To demonstrate the within-subject relationship across conditions, we correlated the σ for the high and low visual intelligibility conditions across subjects. We found a very high correlation (Figures 6A,B) for both the blurred stimuli (*r* = 0.92, *p* < 10^−6^) and the unblurred stimuli (*r* = 0.95, *p* < 10^−7^). This correlation demonstrates that subjects have a consistent level of sensory noise: some subjects have a low level of sensory noise across different stimulus manipulations, while others have a higher level. In our study, the subjects were healthy controls, leading to a modest range of σ across subjects (70–300 ms). Although we fit the model with a restricted range of stimuli (congruent audiovisual words with high or low visual reliability and intelligibility), subjects with high sensory noise might show differences from subjects with low sensory noise across a range of multisensory speech tasks, such as perception of the McGurk effect (Stevenson et al., 2012). In clinical populations, subjects with language impairments (such as those with ASD or dyslexia) might be expected to have higher sensory noise values and a larger variance across subjects.

Next, we examined the average value of σ in different conditions (Figure 6C). Blurring the visual speech should lead to an increase in sensory noise, as the blurred stimuli provide less reliable information about the onset time of the visual speech. Words with lower visual intelligibility should also cause an increase in sensory noise, as the visual speech information is more ambiguous and its onset harder to estimate. As expected, σ was higher for blurred stimuli (mean increase of 10 ms) and low visual intelligibility words (mean increase of 12 ms). A repeated measures ANOVA on the fitted σ values with visual reliability (reliable or blurred) and visual intelligibility (high or low) as factors showed a marginally reliable effect of visual reliability [*F*_(1, 15)_ = 4.51, *p* = 0.051], a main effect of visual intelligibility [*F*_(1, 15)_ = 7.16, *p* = 0.020] and no interaction.

An additional parameter of the CIMS model is *p*_*C* = 1_, which represents the observer's prior belief that audio and visual speech events arise from a common cause. Higher values indicate a higher probability of inferring a common cause (and therefore of responding synchronous). Across all stimulus conditions, subjects' priors were biased toward reporting one cause [Mean *p*_*C* = 1_ = 0.58; *t*-test against 0.5; *t*_(15)_ = 4.47, *p* < 0.001]. A prior biased toward reporting a common cause may be due to the presentation of a single movie clip in each trial and the same talker across trials. Having a high prior for *C* = 1 increases the probability of responding synchronous even for very high asynchronies, leading to the observed behavioral effect of non-zero reported synchrony even at very large asynchronies.

Finally, we examined model parameters that relate to the natural statistics of audiovisual speech. Across all participants, the standard deviation of the common and separate cause distributions were estimated to be σ_*C* = 1_ = 65 ± 9 ms (SEM) and σ_*C* = 2_ = 126 ± 12 ms. For consistency with the literature, we used the stimulus manipulation reference frame and fixed μ_*C* = 1_ at zero, resulting in a fitted value for μ_*C* = 2_ of −48 ± 12 ms (using the physical asynchrony reference frame would result in a value for μ_*C* = 2_ near zero and a positive value for μ_*C* = 1_).

### Experiment 2

Results from the first experiment demonstrated that the CIMS model describes audiovisual synchrony judgments better than the Gaussian model under manipulations of temporal asynchrony, visual blurring, and reduced visual intelligibility. One notable difference between the two models is the use of parameters in the CIMS model that are designed to reflect aspects of the natural statistics of audiovisual speech. If these stimulus parameters are reflective of natural speech statistics, they should be relatively consistent across different individuals tested with the same stimuli. To test this assertion, we fit the CIMS model to an independent set of 21 subjects using the mean values from experiment 1 for the stimulus parameters σ_*C* = 1_, σ_*C* = 2_, and μ_*C* = 2_ (μ_*C* = 1_ remained fixed at zero). We then compared the fits of this reduced CIMS model with the fits from the Gaussian model using the behavioral data from the 21 subjects. In experiment 2, the CIMS model has 7 fewer parameters *per subject* than the Gaussian model (5 vs. 12).

#### Behavioral results and model testing

Overall behavioral results were similar to Experiment 1. The BIC measure favored the CIMS model both for the group (BIC difference: 33.8 ± 1.3; Figure 5C) and in each of the 21 subjects (Figure 5D). A paired *t*-test on the BIC values confirmed that CIMS was the better fitting model [*t*_(20)_ = 25.70, *p* < 10^−15^]. There is a noticeable difference in the average total BIC between experiments. Because the calculation of BIC scales with the number of trials, with 4 trials per subject in Experiment 2 and 12 trials per subject in Experiment 1, the magnitudes will necessarily be larger in Experiment 1 and cannot be directly compared.

The better fit for the CIMS model in this experiment shows that the model is reproducing essential features of synchrony perception with fewer parameters than the Gaussian model. If both models were simply curve-fitting, we would expect the model with more free parameters to perform better. Instead, the CIMS model makes explicit predictions that some parameters should remain fixed across conditions and provides an explanation for the shape of the synchrony judgment data.

## Discussion

The CIMS model prescribes how observers should combine information from multiple cues in order to optimally perceive audiovisual speech. Our study builds on previous examinations of causal inference during audio-visual multisensory integration but provides important advances. Previous work has demonstrated that causal inference can explain behavioral properties of audiovisual spatial localization using simple auditory beeps and visual flashes (Kording et al., 2007; Sato et al., 2007). We use the same theoretical framework, but for a different problem, namely the task of deciding if two speech cues are synchronous. Although the problems are mathematically similar, they are likely to be subserved by different neural mechanisms. For instance, audiovisual spatial localization likely occurs in the parietal lobe (Zatorre et al., 2002) while multisensory speech perception is thought to occur in the superior temporal sulcus (Beauchamp et al., 2004). Different brain areas might solve the causal inference problem in different ways, and these different implementations are likely to have behavioral consequences. For instance, changing from simple beep/flash stimuli to more complex speech stimuli can change the perception of simultaneity (Love et al., 2013; Stevenson and Wallace, 2013). Because multisensory speech may be the most ethologically important sensory stimulus, it is critical to develop and test the framework of causal inference for multisensory speech perception.

The CIMS model shows how causal inference on auditory and visual signals can provide a mechanistic understanding of how humans judge multisensory speech synchrony. The model focuses on the asynchrony between the auditory and visual speech cues because of the prevalence of synchrony judgment tasks in the audiovisual speech literature and its utility in characterizing speech perception in healthy subjects and clinical populations (Lachs and Hernandez, 1998; Conrey and Pisoni, 2006; Smith and Bennetto, 2007; Rouger et al., 2008; Foss-Feig et al., 2010; Navarra et al., 2010; Stevenson et al., 2010; Vroomen and Keetels, 2010). A key feature of the CIMS model is that it is based on a principled analysis of how an optimal observer should solve the synchrony judgment problem. This feature allows it to make predictions (such as the stimulus-level parameters remaining constant across groups of observers) that can never be made by *post-hoc* curve-fitting procedures, Gaussian or otherwise. Hence, the model acts as a bridge between primarily empirical studies that examine subjects' behavior (Wallace et al., 2004; Navarra et al., 2010; Hillock-Dunn and Wallace, 2012) under a variety of different multisensory conditions and more theoretical studies that focus on Bayes-optimal models of perception (Kording et al., 2007; Shams and Beierholm, 2010).

Across manipulations of visual reliability and visual intelligibility, the model fit better than the Gaussian curve-fitting approach, even when it had many fewer parameters. Unlike the Gaussian approach, the parameters of the CIMS model are directly related to the underlying decision rule. These parameters, such as the subject's sensory noise, beliefs about the task, and structural knowledge of audiovisual speech can be used to characterize individual and group differences in multisensory speech perception.

An interesting observation is that the individual differences in sensory noise across subjects (range of 70–300 ms) was much greater than the change in sensory noise within individuals caused by stimulus manipulations (~30 ms). This means that results from only one condition may be sufficient to study individual differences in synchrony perception. In some populations, it is prohibitively difficult to collect a large number of trials in many separate conditions. A measure of sensory noise that is obtainable from only one condition could therefore be especially useful for studying these populations.

### Causal inference predicts features of audiovisual speech synchrony judgments

The CIMS model explains synchrony perception as an inference about the causal relationship between two events. Several features of synchrony judgment curves emerge directly from the computation of this inference process. First, the presence of uncertainty in the sensory system leads to a broad distribution of synchrony responses rather than a single peak near μ_*C* = 1_. When an observer hears and sees a talker, the measured asynchrony is corrupted by sensory noise. The optimal observer takes this noise into account and makes an inference about the likelihood that the auditory and visual speech arose from the same talker, and therefore, are synchronous. This decision process can lead to an overall synchrony judgment curve with a noticeably flattened peak, as observed behaviorally.

Second, the rightward shift (toward visual-leading asynchronies) of the maximal point of synchrony is explained by the natural statistics of audiovisual speech coupled with noise in the sensory system. Because the mean of the common cause distribution (μ_*C* = 1_) is over the visual-leading asynchronies, small positive asynchronies are more consistent with a common cause than small negative asynchronies. This feature of the synchrony judgment curve is enhanced by the location of the *C* = 2 distribution at a physical asynchrony of 0 ms.

### What about the temporal binding window and the mean point of synchrony?

The Gaussian model is used to obtain measures of the temporal binding window and mean point of synchrony in order to compare individuals and groups. In our formulation of the CIMS model, we introduced the Bayes-optimal synchrony window. This synchrony window should not be confused with the temporal binding window. The temporal binding window is predicated on the idea that observers have access to the physical asynchrony of the stimulus, which cannot be correct: observers only have access to a noisy representation of the world. The CIMS model avoids this fallacy by defining a synchrony window based on the observer's noisy measurement of the physical asynchrony. The predicted synchrony reports from the CIMS model therefore relate to the probability that a *measured* asynchrony will land within the Bayes-optimal window, not whether a *physical* asynchrony is sufficiently small. This distinction is a critical difference between the generative modeling approach of the CIMS model and the curve-fitting approach of the Gaussian model.

In the CIMS model, the shape of the behavioral curve emerges naturally from the assumptions of the model, and is a result of interactions between all model parameters. In contrast, the mean point of synchrony in the Gaussian model defines a single value of the behavioral data. This poses a number of problems. First, the behavioral data often show a broad plateau, meaning that a lone “peak” mean point of synchrony fails to capture a prominent feature of the behavioral data. Second, the location of the center of the behavioral data is not a fixed property of the observer, but reflects the contributions of prior beliefs, sensory noise, and stimulus characteristics. By separately estimating these contributions, the CIMS model can make predictions about behavior across experiments.

### Modifications to the gaussian model

The general form of the Gaussian model used in this paper has been used in many published studies on synchrony judgments (Conrey and Pisoni, 2006; Navarra et al., 2010; Vroomen and Keetels, 2010; Baskent and Bazo, 2011; Love et al., 2013). It is possible to modify the Gaussian model used in this paper to improve its fit to the behavioral data, for instance by fitting each side of the synchrony judgment curve with separate Gaussian curves (Powers et al., 2009; Hillock et al., 2011; Stevenson et al., 2013). In the current study, the Gaussian model required 12 parameters per subject; fitting each half of the synchrony judgment curve would require 20 parameters per subject, more than twice the number of parameters in the CIMS model. Additionally, the CIMS model required only 4 parameters to characterize changes across experimental conditions. Although researchers may continue to add more flexibility to the Gaussian model to increase its fit to the behavioral data (Stevenson and Wallace, 2013), the fundamental problem remains: the only definition of model goodness is that it fits the behavioral data “better.” In the limit, a model with as many parameters as data points can exactly fit the data. Such models are incapable of providing a deeper understanding of the underlying properties of speech perception because their parameters have no relationship with underlying cognitive or neural processes.

Researchers using the Gaussian model could also try to reduce the number of free parameters by fixing certain parameters across conditions, or using values estimated from independent samples. This approach is necessarily *post-hoc*, providing no rationale about which parameters should remain fixed (or allowed to vary) before observing the data.

## Conclusion

The CIMS model affords a quantitative grounding for multisensory speech perception that recognizes the fundamental role of causal inference in general multisensory integration (Kording et al., 2007; Sato et al., 2007; Schutz and Kubovy, 2009; Shams and Beierholm, 2010; Buehner, 2012). Most importantly, the CIMS model represents a fundamental departure from curve-fitting approaches. Rather than focusing on mimicking the shape of the observed data, the model suggests how the data are generated through a focus on the probabilistic inference problem that underlies synchrony perception. The model parameters thus provide principled measures of multisensory speech perception that can be used in healthy and clinical populations. More generally, causal inference models make no assumptions about the nature of the stimuli being perceived, but provide strong predictions about multisensory integration across time and space. While the current incarnation of the CIMS model considers only temporal asynchrony, the theoretical framework is amenable to other cues that may be used to make causal inference judgments during multisensory speech perception, such as the spatial location of the auditory and visual cues, the gender of the auditory and visual cues, or the speech envelope.

### Conflict of interest statement

The authors declare that the research was conducted in the absence of any commercial or financial relationships that could be construed as a potential conflict of interest.
